# Tachycardia Initiated by One to Two Response and Terminated by ICD Shocks

**Published:** 2010-12-26

**Authors:** Claude S Elayi, Gustavo X Morales, Bahram Kakavand, Jignesh S Shah

**Affiliations:** 1 Gill heart Institute, University of Lexington, Lexington, Kentucky; 2 University of Lexington, Lexington, Kentucky; 3 Arrhythmia Associates, Mumbai, India

**Keywords:** One to Two Response, ICD Shocks

## Case presentation

**Figure 1:**
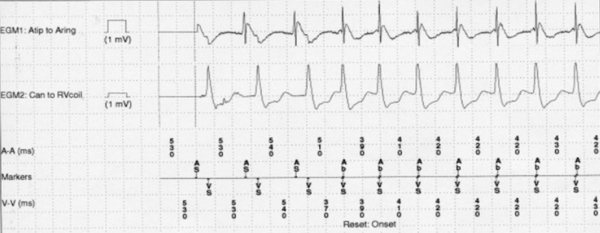
Example of stored EGM showing initiation of tachycardia. The channels from the top to the bottom represent the atrial electrograms, the ventricular electrograms and the ICD markers. See text for description.

A 58 year-old male was referred to our institute for recurrent palpitations followed by ICD shocks. The patient had a history of ischemic cardiomyopathy (EF 25%) and had received a dual-chamber ICD two years earlier for primary prevention of sudden cardiac death. The device interrogation showed multiple episodes of tachycardia and anti-tachycardia pacing (ATP), which frequently terminated the tachycardia. However, occasional failure to terminate the tachycardia or immediate re-initiation within the redetection window led to ICD shocks. A typical episode of tachycardia with its initiation is shown in Figure 1.

Device interrogation showed the onset of tachycardia with a near-simultaneous atrial and ventricular activity (fourth beats). Such an onset of tachycardia with less atrial beats than ventricular beats rules out atrial tachycardia. Device interrogation also revealed multiple sequences of ATP that entrained the tachycardia with a VAV response further ruling out atrial tachycardia. The ventricular electrogram morphology during sinus rhythm was similar to that during tachycardia, and the 12-lead EKG demonstrated identical wide QRS morphology during sinus rhythm and during tachycardia (typical left bundle block branch pattern at baseline). The similarity in QRS morphology (and ventricular electrogram) during sinus rhythm and tachycardia could be consistent with bundle branche reentry VT as those arrhythmias are typically seen in patients with severe cardiomyopathy (like our patient). However, the tachycardia initiation with simultaneous V and A makes this diagnosis unlikely.  Based on these observations, the two differential diagnoses are:

-junctional ectopic tachycardia (JET) that starts with V (4th beat) and conducts retrogradely to A (4th beat).

-typical slow fast atrioventricular nodal reentrant tachycardia (AVNRT) that initiates with A (3rd beat) leads to V (3rd beat) through the fast pathway and gives a second V (4th beat) through the slow pathway (1:2 response).

**Figure 2:**
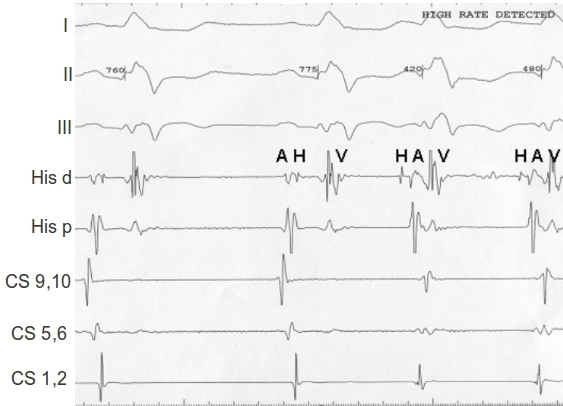
Spontaneous initiation of the tachycardia during the EP study.  The channels from the top to the bottom represent: ECG leads I, II and III; HIS distal and proximal; coronary sinus (CS) decapolar catheter with respectively proximal (9,10), mid (5,6) and distal (1,2) shown here.

The patient underwent an electrophysiology study after discontinuing the beta blockers for 5 days. The baseline intracardiac intervals were unremarkable. The mode of tachycardia initiation   was either spontaneous (see Figure 2) or after single atrial premature depolarization (APD) with a single AH jump.  The 12-lead EKG once again confirmed the similarity in the wide QRS morphology during sinus rhythm and tachycardia (Figure 2).

Further EP maneuvers were performed to differentiate JET and AVNRT as recently reported [1]. APD delivered prior to His repeatedly terminated the tachycardia, which would be unlikely in JET.  However, an APD delivered prior to His during AVNRT will activate the AV nodal fast pathway antegradely making it refractory to retrograde conduction, thereby terminating AVNRT.  In addition, an APD delivered during His refractoriness modified the next His, A and V, an unlikely observation with JET, but again consistent with AVNRT. Because the AV nodal slow pathway conduction is not involved in initiation or maintenance of JET, an APD timed to junctional depolarization should be no perturbation of the subsequent JET beats. Hence, a change occurring in the subsequent tachycardia beat (such as advancement, delay, or termination of tachycardia) excludes JET and confirms the implication of the slow AV nodal pathway for anterograde conduction (AVNRT).

Based on these findings, the tachycardia was thus diagnosed as AVNRT. The slow pathway area was successfully targeted with one RF application to render the tachycardia non-inducible.  No arrhythmia has been observed on the ICD telemetry for more than a year.

## Discussion

**Figure 3:**
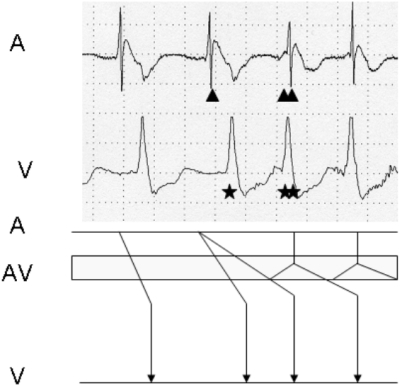
Explanation of tachycardia in Figure 1 using a ladder diagram

In the case presented here, a sinus beat led to 1:2 response with conduction via the fast pathway generating the first QRS* followed by conduction over the slow pathway leading to the second QRS** and induction of AVNRT (Figure 3).

An atrial extrasystole causing 1:2 response is an uncommon phenomenon that has been previously described [2]. The mechanism of the tachycardia commonly reported with this phenomenon is a sinus rhythm with a fast ventricular rate twice that of the atrial rate (also called non-reentrant tachycardia). Initiation of AVNRT following a 1:2 response during programmed atrial stimulation has been reported [3]. However, in our patient it was a non-premature sinus beat which led to 1:2 response and initiated the tachycardia. This is most likely due to changes in the autonomic tone affecting the electrophysiological properties of the slow and fast pathways leading to 1:2 response and induction of tachycardia during sinus rhythm. Interestingly the sinus node did not seem affected by the change in the autonomic tone. This case demonstrates the differential effect of changes in the autonomic tone on sinus node and the dual AV node pathways. In addition, this case serves as a reminder that a precise mechanism for SVT can sometimes be gleaned from the ICD tracings alone after systematic examination of all intracardiac electrograms. The device interrogation can help in recognizing and treating easily some arrhythmias in patients with structural heart disease.
